# A case of BIA-ALCL in which postoperative chest wall recurrence was highly suspected: the third reported case of BIA-ALCL in Japan

**DOI:** 10.1186/s40792-024-01996-6

**Published:** 2024-08-23

**Authors:** Wakako Tajiri, Ryo Shimamoto, Yutaka Koga, Junji Kawasaki, Makiko Higuchi, Yoshiaki Nakamura, Yumiko Koi, Chinami Koga, Hideki Ijichi, Ilseung Choi, Youko Suehiro, Kenichi Taguchi, Eriko Tokunaga

**Affiliations:** 1 Department of Breast Oncology, National Organization Kyushu Cancer Center, 3-1-1 Notame, Minami-Ku, Fukuoka, 811-1395 Japan; 2 Department of Plastic Surgery, National Organization Kyushu Cancer Center, 3-1-1 Notame, Minami-Ku, Fukuoka, 811-1395 Japan; 3 Department of Pathology, National Organization Kyushu Cancer Center, 3-1-1 Notame, Minami-Ku, Fukuoka, 811-1395 Japan; 4 Department of Hematology and Cell Therapy, National Organization Kyushu Cancer Center, 3-1-1 Notame, Minami-Ku, Fukuoka, 811-1395 Japan

**Keywords:** BIA-ALCL, CD30, ALK (anaplastic lymphoma kinase), Breast cancer, Breast implant

## Abstract

**Background:**

Breast implant-associated anaplastic large-cell lymphoma (BIA-ALCL) is a rare malignancy. Many cases of BIA-ALCL are identified based on the presence of late-onset effusion and/or masses. Importantly, the United States Food and Drug Administration noted that in all cases diagnosed in patients with textured implants, the patients either had a history of mixed implantation of smooth and textured devices or no clinical history was supplied for review. In Japan, the first case of BIA-ALCL was reported in 2019, and we encountered the third case in Japan in December 2021. There have been a total of five cases of BIA-ALCL previously reported at Japanese academic conferences (Japan Oncoplastic Breast Surgery Society. http://jopbs.umin.jp/medical/index.html), of which only the first case has been published. Unlike the first case, this patient had clinical features that were highly suggestive of the postoperative chest wall recurrence of breast cancer, with a mass and rash on the skin.

**Case presentation:**

The patient was a 45-year-old woman who had undergone breast reconstruction after breast cancer surgery of the right breast 8 years previously. The patient presented with a mass and skin rash inside the inframammary area, and we suspected a damaged silicone breast implant (SBI) or chest wall recurrence. We examined the mass by a core needle biopsy and made a pathological diagnosis of BIA-ALCL. Imaging findings suggested internal thoracic lymph node swelling and lymphoma infiltration beyond the capsule but no metastatic lesions (cStage III). After en bloc resection of the SBI and lymphoma, adjuvant systemic therapy was performed.

**Conclusion:**

We encountered the third case of BIA-ALCL in Japan. This was a case with clinically advanced stage of disease; however, the BIA-ALCL was found to be in remission.

## Background

Breast implant-associated anaplastic large-cell lymphoma (BIA-ALCL) is a rare malignancy that was classified as a novel lymphoma by the World Health Organization in 2016 [1]. The National Comprehensive Cancer Network (NCCN) established evidence-based consensus guidelines for the diagnosis and treatment of BIA-ALCL [2]. Many cases of BIA-ALCL have been found with late-onset effusion and/or masses. Eight to 24% of patients are diagnosed with an associated palpable mass, while 4–12% are diagnosed with lymphadenopathy [3]. Ultrasound is useful for detecting effusion (84–75%) or masses (46–100%) associated with BIA-ALCL [4]. Ultrasound is also useful for observing implant displacement and deformity associated with BIA-ALCL. Pathological evaluations, such as fine needle aspiration (FNA) cytology, fluid cytology, and a biopsy of the mass, are necessary for the diagnosis. BIA-ALCL can be diagnosed when specimens are positive for CD30 and negative for anaplastic lymphoma kinase (ALK) protein [5].

Importantly, the United States Food and Drug Administration noted that in all cases diagnosed in patients with texture implants, the patients either had a history of mixed implantation of smooth and textured devices or no clinical history available for review. To date, there have been no confirmed cases of BIA-ALCL in patients with only smooth devices [6–8].

BIA-ALCL is still uncommon, and its risk factors and prognosis are still unclear. In Japan, the first case was reported in 2019, and the third case was encountered in December 2021. Unlike the first case, this patient had clinical features that were highly suggestive of postoperative chest wall recurrence of breast cancer, with a mass and rash on the skin.

In this report, we describe the third case of BIA-ALCL, which was diagnosed based on the evaluation of a nodule that was suspected to be a local recurrence of breast cancer. We previously published a brief report on the pathological findings of this case in the Japanese journal regarding the pathology in Japanese [9]. We herein report the detailed clinical course of the third case of BIA-ALCL reported in Japan.

## Case presentation

The patient was a 45-year-old woman who had undergone right total mastectomy, sentinel node dissection, and tissue expander insertion for right breast cancer 10 years previously. The histopathological diagnosis was invasive carcinoma, mucinous carcinoma, pT1cN0M0 stage IA, and the patient underwent adjuvant endocrine therapy for 5 years. She did not undergo radiation therapy or chemotherapy.

Replacement with textured implants was performed 6 months after breast cancer surgery. At 1 year and 4 months after the first breast reconstruction, she requested reconstruction again, and then underwent a second replacement with a textured surface silicone breast implant (SBI, Allergan/410 MM280 g) and nipple and areola surgery (NAC reconstruction). Eight years after the second SBI replacement, the patient noticed a palpable and painful nodule with a rash and skin eruption in the right inferior inner chest area and visited the plastic surgery clinic where she had received reconstructive surgery. Local recurrence of breast cancer was suspected; therefore, the patient consulted our department for a detailed examination and treatment.

**Fig. 1:**
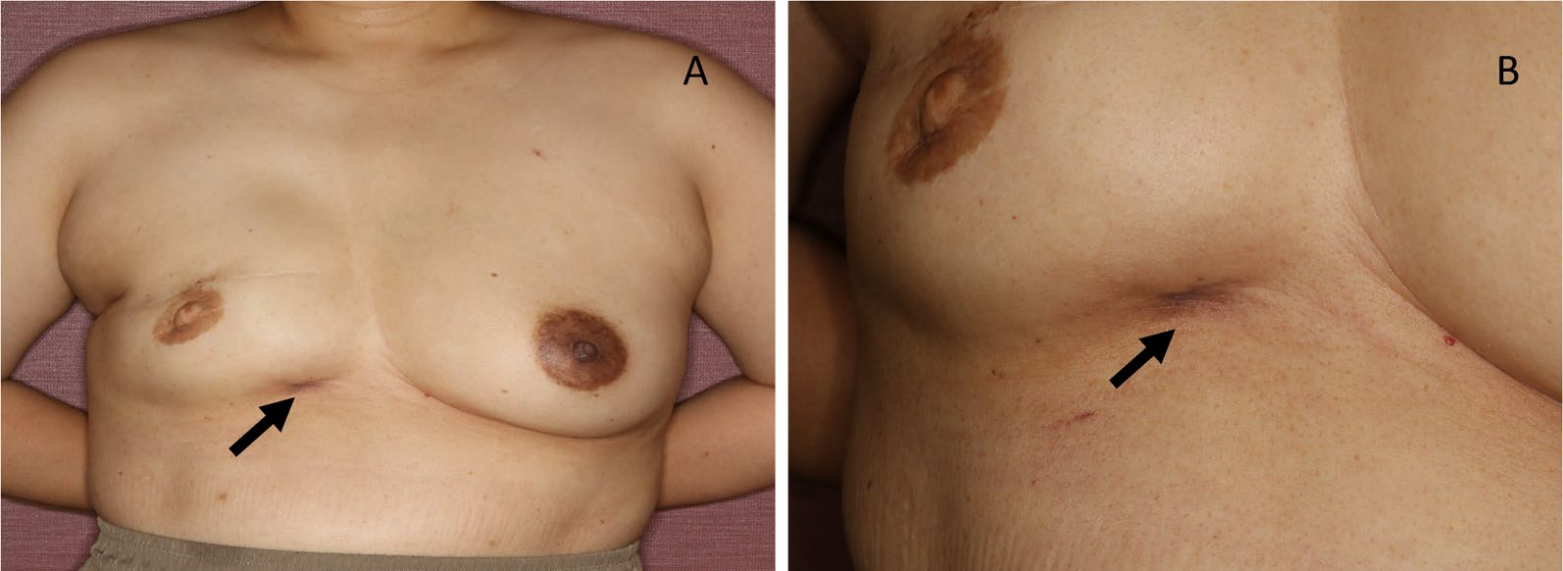
Appearance of the right breast. A palpable mass with a skin rash and dimple was observed in the inner inframammary area of the right breast (arrow). **A** Distant view, **B** close view

**Fig. 2:**
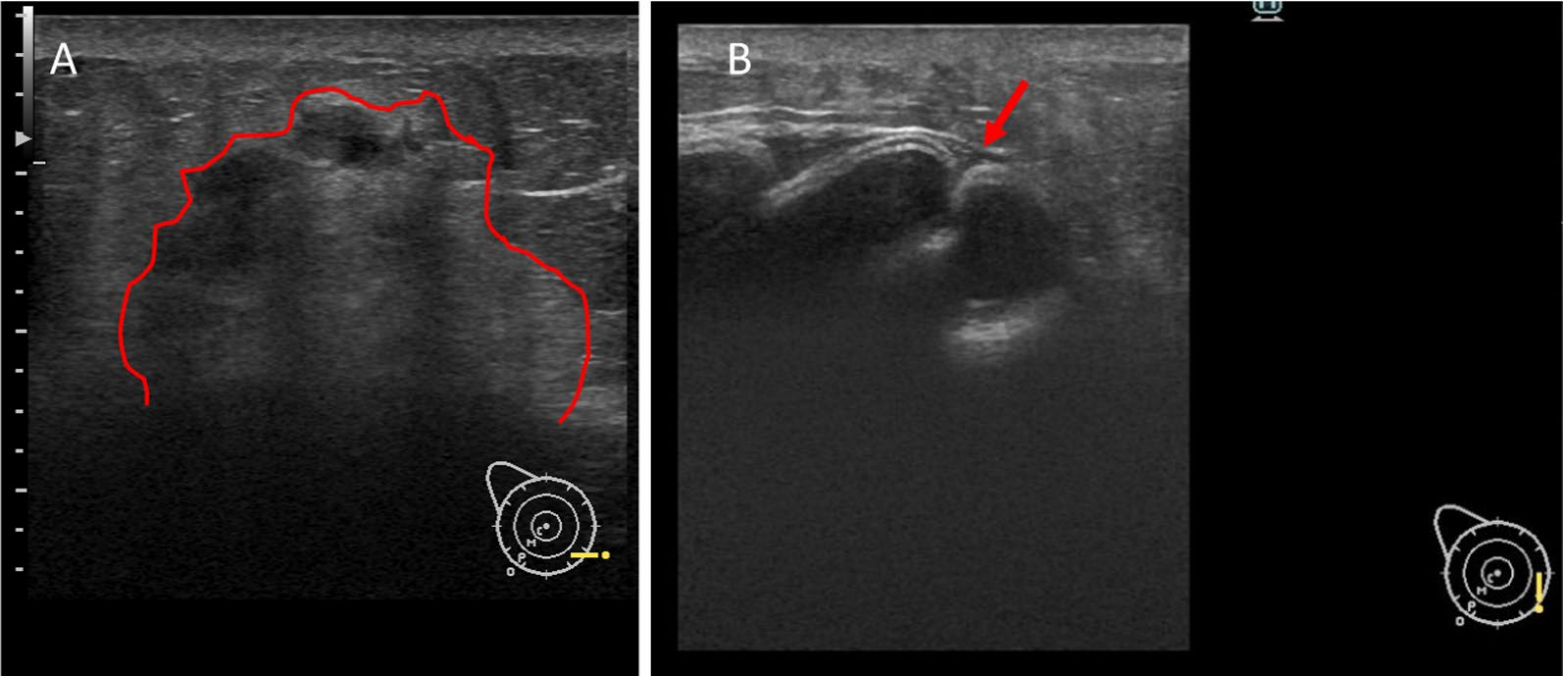
Ultrasound findings. **A** A hypoechoic irregular mass with a surrounding hyperechoic area was recognized in the inner inframammary area of the right breast. The outline is shown in red line **B**. The implant shell was deformed and a wavy pattern was recognized (red arrow)

Immobile mass and redness of the skin were recognized on the caudal side of the right inframammary fold (Fig. 1). Ultrasound showed a hypoechoic irregular mass (7.7 × 6.1 × 4.2 mm) with a surrounding hyperechoic area. The border of the mass was vague, the inner echo level was heterogeneous, and inner blood flow was observed. There was no seroma around the implants. The implant shell was deformed and presented a wavy pattern; however, no obvious damage to the implant was observed (Fig. 2). A review of the medical records showed that implant deformation had started 2 years before this point.

**Fig. 3:**
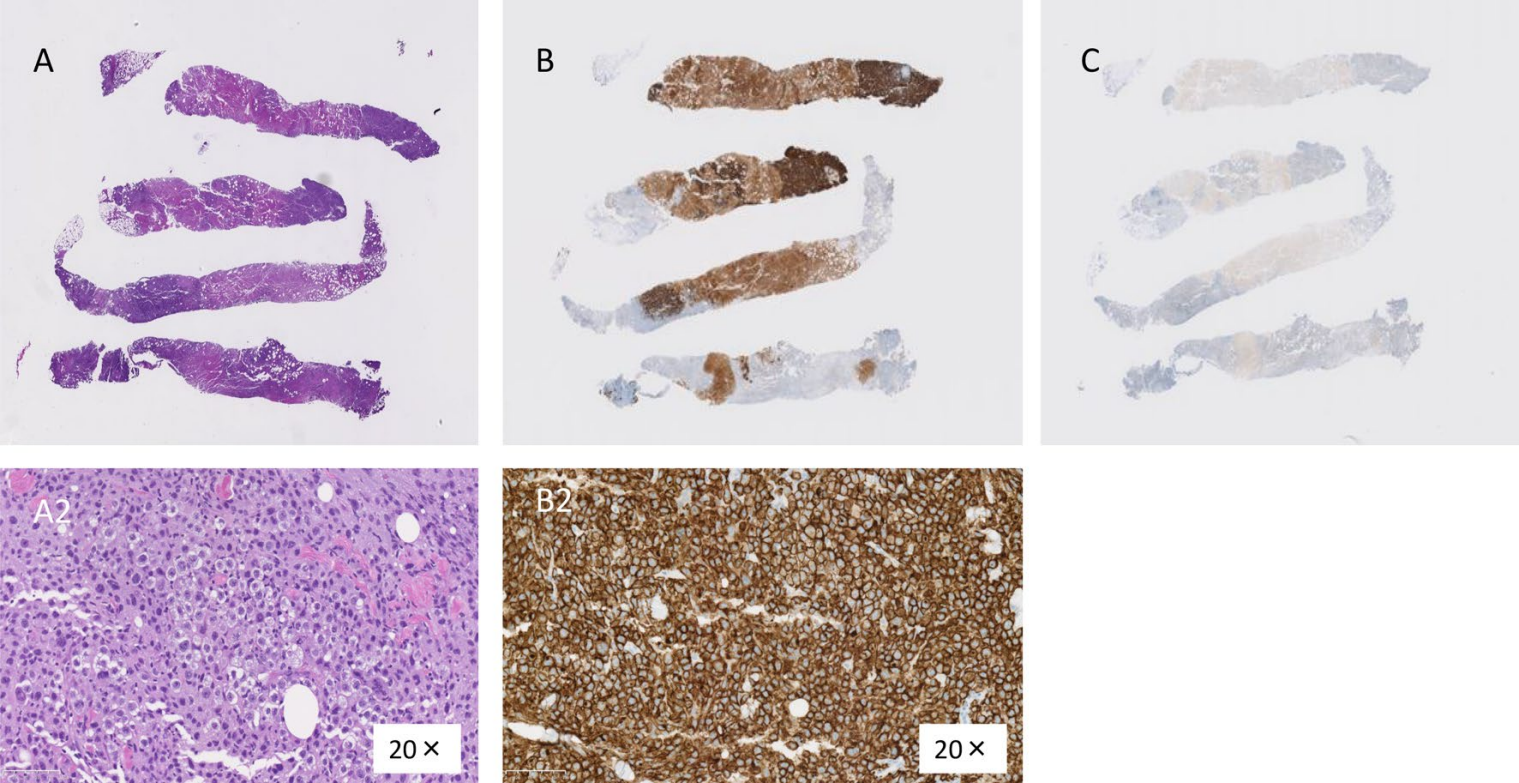
Microscopic findings of the core needle biopsy. **A** Hematoxylin eosin staining. There were many atypical cells with extensive necrotic tissue. (A2) 20×. **B** Immunohistochemistry was positive for CD30. (B2) 20×. **C** Immunohistochemistry was negative for ALK

**Fig. 4:**
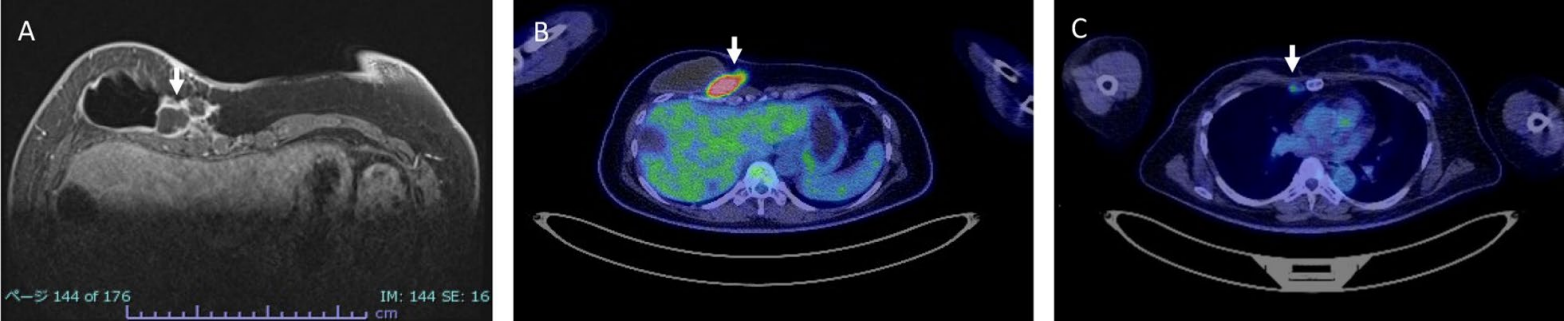
Preoperative imaging. MRI and PET–CT findings. **A** MRI. An irregular mass enhanced only on the margin was recognized in the inner side of the implant of right breast (white arrow). **B**, **C** FDG-PET–CT. **B** A mass with high FDG accumulation (SUVmax = 41.54) at the same position as on MRI (white arrow). **C** An internal mammary lymph node was swollen and showed mild FDG accumulation (SUVmax = 2.82), which suggested metastasis (white arrow)

**Fig. 5:**
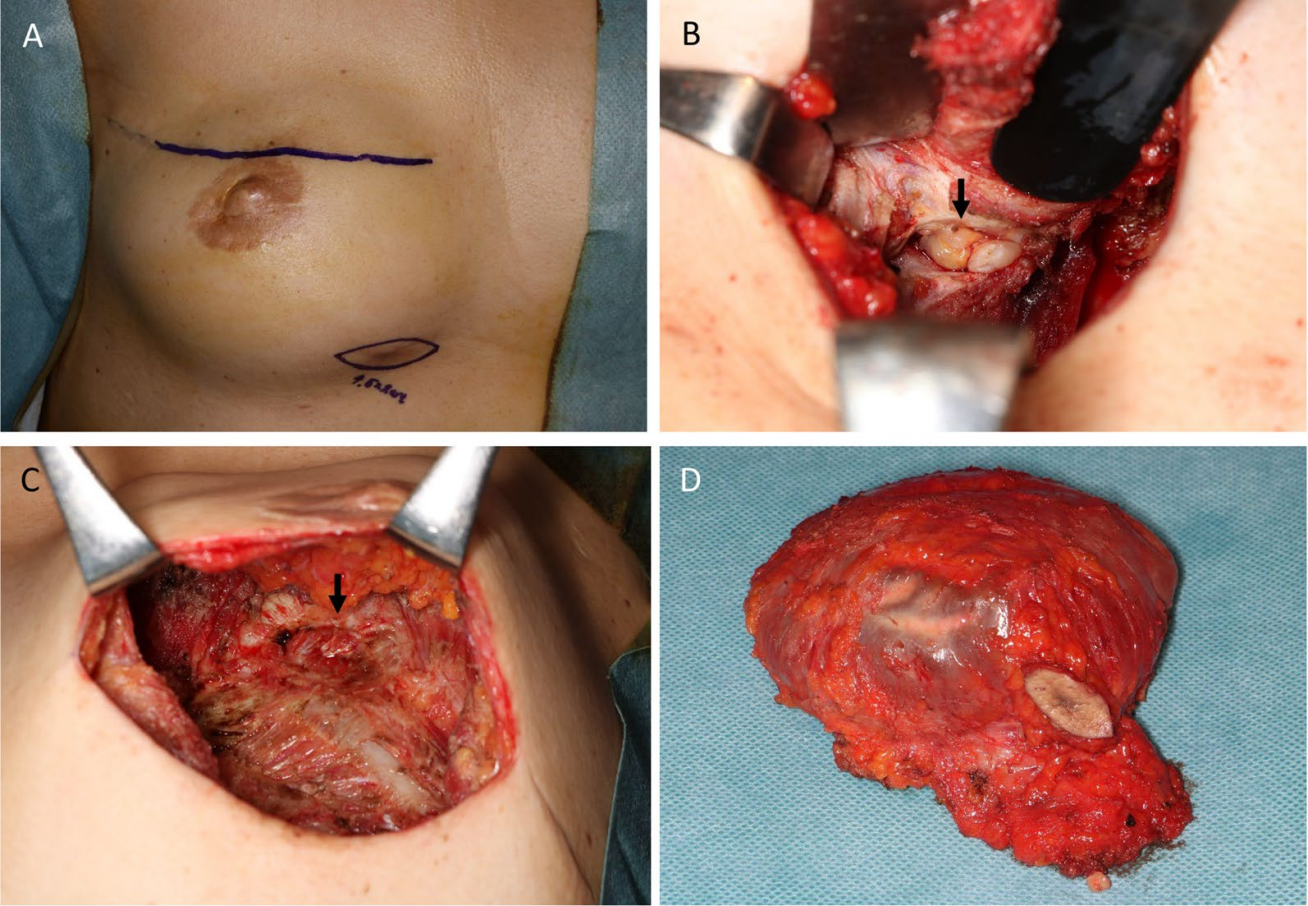
Surgical findings. **A** Two skin incisions were added in order to remove the implant and lymphoma with a clear margin. One incision was made on the implant surgery scar, and another was made over the lymphoma nodule. **B** Multiple lymphoma nodules were observed in an encapsulated space (arrow). **C** The appearance after the total removal of the implant and lymphoma. The right pectoralis major muscles were partly resected (arrow). **D** The implant and lymphoma were resected en bloc

**Fig. 6:**
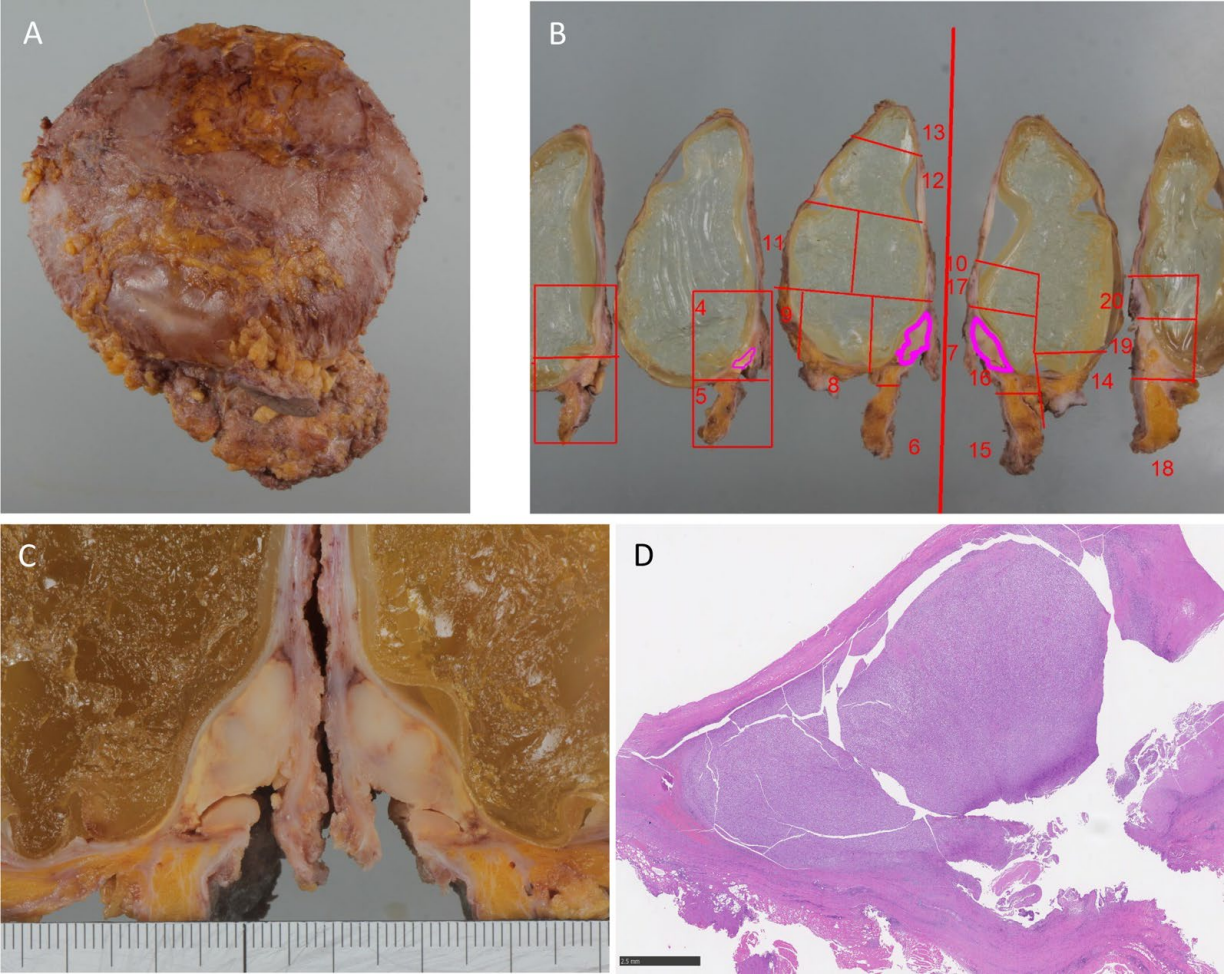
Histopathological findings. **A** Appearance of the resected implant and the lymphoma after fixation with formalin. **B** Macro image. The lymphoma lesion was 22 × 9 × 12 mm in size, located outer side of the implant, surrounded by soft tissue (purple line). **C** Enlarged photo of lymphoma tissue and part of the implant. **D** Pathological images at low magnification

The mass was considered to be a chest wall recurrence of breast cancer, and a core needle biopsy was performed. A pathological examination showed atypical cells with extensive necrosis demonstrated by hematoxylin and eosin (HE) staining. On immunohistochemistry, the mass was CD30-positive and ALK-negative. Accordingly, a diagnosis of BIA-ALCL was made (Fig. 3). The histology obtained by a needle biopsy was distinct from that of primary breast mucinous carcinoma, and the pathologist considered malignant lymphoma first and foremost rather than breast cancer [7]. After obtaining a histological diagnosis, MRI revealed an irregular mass with enhancement only at the marginal area, and invasion of the pectoralis major muscle was suspected. As well as ultrasounds, there was no obvious seroma around the implants. 18-FDG positron emission tomography/computed tomography (PET/CT) showed increased uptake in the mass (SUVmax 41.54), suggesting invasion of the pectoralis major muscle. The internal mammary lymph node was swollen, suggesting lymph node metastasis. However, no obvious distant metastases were observed (Fig. 4). The preoperative staging was cT4N1M0 stage III (http://jopbs.umin.jp/medical/guideline/docs/BIA-ALCL4-5_20220218.pdf). During surgery, SBI and lymphoma were removed en bloc. The lymphoma, which was originally thought to be one lump, was actually composed of multiple nodules of lymphoma in an enclosed space (Fig. 5). The pathological examination revealed a lymphoma that was located outside the capsule and had invaded the pectoral muscle. The lymphoma measured 22 × 9 × 12 mm. The lesion was composed of a proliferation of atypical lymphoid cells with enlarged nuclei, occasional prominent nucleoli, and abundant clear cytoplasm, associated with massive necrosis. The postoperative histopathological diagnosis was BIA-ALCL, which was found to be CD30-positive and ALK-negative by IHC. These findings were consistent with the needle biopsy findings. The surgical margin was free of lymphoma cells (Fig. 6).

Adjuvant systemic therapy with six cycles of A-CHP (brentuximab vedotin, cyclophosphamide, doxorubicin, and prednisolone) was administered at the Department of Hematology in our hospital. Total remission was achieved with chemotherapy alone. After chemotherapy, the swollen internal mammary lymph node preoperatively suspected to metastatic node has disappeared on imaging. Therefore, radiotherapy was not done. The patient is currently doing well.

## Discussion

We report the third case of BIA-ALCL in Japan, which occurred 8 years after breast reconstruction surgery. The patient was diagnosed with a mass that was initially suspected to be a local recurrence (chest wall recurrence). The mass was diagnosed as BIA-ALCL via core needle biopsy, and a detailed examination revealed that the patient had clinical stage III disease.

Between 2019 and 2022, five cases of BIA-ALCL were reported in Japan (http://jopbs.umin.jp/index.html). Although the number of patients with BIA-ALCL reported in Asia is small, reports are increasing [10–13]. Most cases of BIA-ALCL present with a seroma. However, the presence of a breast and/or axillary mass is rare [14]. The present case, which was diagnosed based on the detection of a palpable mass, was also at a higher stage in comparison to other reports [6, 7].

Although a method for early detection has not yet been established, early detection of BIA-ALCL is important [2]. Implant deformity may be a sign of BIA-ALCL. The first case in Japan, reported in 2019, showed a small amount of effusion around the implant and shape changes in the outer shell of the implant on ultrasound [15]. Although the mass was the initial reason for discovery in our case, an ultrasound examination revealed wavy deformation of the outer contour of the implant proximal to the mass. In addition, the implant deformity had begun 2 years prior to the diagnosis of BIA-ALCL in this case, and the degree of corrugation became stronger over time. Implant deformity is suggestive of capsular disease, and it has been reported that capsular disease of breast implants can cause late seromas or masses [16]. Late seroma and mass formation have been observed even in early BIA-ALCL and may be associated with the occurrence of capsular disease and BIA-ALCL. However, to our knowledge, no reports have suggested that complete removal of the capsule in asymptomatic patients reduces the risk of developing BIA-ALCL [17].

Chest wall invasion has been reported to be a poor prognostic factor [18]. As a treatment strategy, if the clinical stage is stage II or higher, multidisciplinary treatment as well as surgery is recommended [5]. It is strongly recommended that breast reconstruction be delayed after BIA-ALCL treatment, especially in stage II Acw-IV cases with chest wall invasion, and routine imaging examinations are considered necessary [19]. It is recommended that reconstruction be delayed for at least 5 years after the completion of multidisciplinary treatment [20]. The present patient wanted re-reconstruction; however, she understood the risk after repeated discussions between the patient and the medical staff. The patient was carefully monitored.

In terms of pathogenesis, BIA-ALCL exhibits a specific pattern of genetic alterations. The pathogenesis of BIA-ALCL, which involves genetic predisposition, mainly includes JAK-STAT, DNA methyltransferase 3 alpha (DNMT3A) mutation, tumor protein p53 (TP53) mutation, programmed cell death 1 ligand 1 (PD-L1) chromosomal copy number aberrations (CNAs), chromosome 20q loss, and the overexpression of carbonic anhydrase 9 (CA9). Genetic susceptibility is an important factor for the occurrence and development of BIA-ALCL [14]. No genetic alterations were detected in the present case.

In conclusion, we reported the third case of BIA-ALCL in Japan, which was initially identified based on the detection of a mass. We believe that it is important to keep BIA-ALCL in mind as a differential diagnosis when a mass is observed in a breast cancer patient who has undergone reconstruction with an SBI, irrespective of the presence of effusion around the SBI or changes on the surface of the SBI.

## Conclusions

This was the third case of BIA-ALCL recognized in Japan. This case occurred 8 years after breast reconstruction surgery, and the stage of the disease was the most advanced reported so far in Japan. The patient required multidisciplinary treatment, not only surgery but also systemic therapy. The postoperative course was good, and the patient is still in remission.

## Data Availability

Not applicable.

## Abbreviations

BIA-ALCL: Breast implant-associated anaplastic large-cell lymphoma
FNA: Fine needle aspiration
ALK: Anaplastic lymphoma kinase
SBI: Silicone breast implant
HE: Hematoxylin and eosin
MRI: Magnetic resonance imaging
PET/CT: 18-FDG positron emission tomography/computed tomography
SUV: Standard uptake value
CHP: Brentuximab vedotin, cyclophosphamide, doxorubicin, and prednisolone
DNMT3A: DNA methyltransferase 3 alpha
TP53: Tumor protein p53
PD-L1: Programmed cell death 1 ligand 1
CNAs: Copy number aberrations
CA9: Carbonic anhydrase 9
